# Ear measurement of temperature is only useful for screening for fever in an adult emergency department

**DOI:** 10.1186/s12873-018-0202-5

**Published:** 2018-12-03

**Authors:** Christian Backer Mogensen, Malene Bue Vilhelmsen, Johanne Jepsen, Lilian Keene Boye, Maiken Hjuler Persson, Florence Skyum

**Affiliations:** Emergency Department, Sygehus Sønderjylland, Kresten Philipsensvej 15, 6200 Aabenraa, Denmark

**Keywords:** Fever, Temperature measurement, Tympanic, Rectal temperature

## Abstract

**Background:**

A new generation of ear thermometers with preheated tips and several measurements points should allow a more precise temperature measurement. The aim of the study was to evaluate if the ear temperature measured by this ear thermometer can be used to screen for fever and whether the thermometer is in agreement with the rectal temperature and if age, use of hearing devices or time after admission influences the temperature measurements.

**Methods:**

Open cross-sectional clinical single site study patients, > 18 years old, who were acutely admitted to the short stay unit at the ED. A sample size of 99 patient per subgroup was recruited as random convenience series. As ear thermometer Braun Thermoscan Pro 4000® and as rectal thermometer Omron Flex Temp Smart ® was used. For different cut off of temperature the AUC was calculated and Bland-Altman analysis for calculation of 95% limits of agreement with rectal temperature, with subgroup analysis concerning age, time span from admission time and use of hearing aid.

**Results:**

Among 599 patients the sensitivity to detect fever with an ear thermometer varied between 68 and 70% with AUC from 0.88–0.97. If the ear temperature was ≥37.5 oC, the sensitivity to detect patients with ≥38.0 oC rectally was 95% which raised to 100% for a rectal temperature of ≥38.3 oC. For the ear thermometer’s ability to determine the exact temperature the 95% limits of agreement were +/− 0.8 oC. with no influence from age, duration of hospital stay or hearing aids.

**Conclusion:**

The examined ear thermometer is able to detect fever, defined as ≥38 oC rectally in an adult ED population by using an ear cut-point of 37.5 oC, but not to measure the exact temperature. Used in this way around a fifth of the patients will still be in need of a rectal temperature measurement, but less than 5% with fever ≥38.0 oC will remain undetected and none with fever ≥38.3 oC. Age, admission time and use of hearing aid did not influence the temperature measurements.

**Trial registration:**

Clinical Trials: ID NCT02977481, date 11/18/2016.

## Background

Fever is important information in the diagnosis of acute infections. The absence of fever might reassure the clinician that severe infection is less likely and thus create potentially dangerous situations if the measurement of temperature is based on inadequate methods [1]. Measurement of body temperature by use of ear thermometers has been widely applied in Danish hospitals and worldwide, as this method is faster and more user and patient friendly than the use of rectal measurements [2, 3]. Rectal temperature is, however, considered the gold standard and several reviews have warned against the first generations of tympanic membrane (TM) thermometers for hospital use [1, 4, 5].

These reviews included a range of first-generation technologies. A new generation of ear thermometers with preheated tips and ability to detect the highest temperature among several measurements within the ear canal should allow a more precise temperature measurement [3]. Some studies recommend replacing rectal measurement with the new generation of TM thermometers [3, 6]. However, these studies are of limited size, not analyzed with the recommended methods or implemented as feasibility or efficacy studies and not in clinical settings. Three clinical studies have been performed on adults comparing the new generation TM thermometers with rectal measurements showing conflicting results. The first study from a surgical ward recommended the use [3] while the second study from an emergency department (ED) found a cut-off of 37.5 °C feasible for screening purposes [7] and the third study, also from an ED, did not recommend the new devices for clinical use [8]. These studies have not analyzed whether the results of the TM measurements were dependent on age, time after arrival from surroundings with a different temperature or use of hearing aids which are all factors that might influence the measurements.

The high patient turn-over in the ED, with an intensive workload for the staff and often lack of privacy, complicates the use of rectal thermometers and many ED have changed to TM measurements despite the lack of evidence [8]. Since the majority of patients in the ED do not have fever and since it is highly important that the few febrile patients are identified, a high sensitivity and negative predictive value of a normal temperature measured by tympanic methods is required, at least above 90% for both values [9].

Acknowledging the conflicting results of the few ED studies there is still a need to examine the usability of these new generations of TM thermometers for adult patients in an ED setting. The aim of the present cross-sectional ED study was to evaluate:

First, if the ear temperature measured with a tip preheated multi-spot tympanic membrane temperature device can be used as a screening measure for fever and to determine at which cut-point a sensitivity higher than 90% can be reached, secondly to analyze if the TM thermometer is in agreement with the rectal temperature within a clinically acceptable range of maximum +/− 0.5 °C [7, 10] and finally if certain conditions like age, the use of hearing devices or time after admission influences the exact temperature measurements.

## Methods

### Study design

This study was an open cross-sectional clinical single site study evaluating the ear thermometer as a diagnostic test for fever and the limits of agreement with the exact rectal temperature. The data collection was planned before the study commenced, i.e. a prospective study design.

### Setting

The Hospital of Southern Jutland, Denmark provides acute medical services for a mixed urban and rural population of around 250.000 people. The Emergency Department has a short stay unit providing care for the first 48 h of admission for patients more than 18 years old, with internal, surgical or orthopedic complaints and receives annually 16.000 acute admissions.

### Study population

The study population was medical and surgical patients, more than 18 years old, who were acutely admitted to the short stay unit at the ED. We excluded patients, who had no rectum, were isolated, pregnant or unable to provide an informed written consent due to language problems or mental incompetence.

A sample size of 99 patients was calculated to be able to detect a difference of 0.3 °C between the two thermometers with a standard deviation of 0.65 °C, a power of 90% and a significance level of 95%, assuming normal distribution. To allow for analysis of 5 subgroups (age, time of arrival and hearing aid) we aimed at collecting at least 500 patients. The obtained data were for all subgroups normal distributed and the standard deviation around 0.6 °C.

The patients were recruited as random convenience series by a research assistant in daytime on randomly selected days and both new admissions, as well as patients who had been admitted up to 24 h earlier, were asked to participate. The patients were only allowed to participate once during their admission.

Eligible patients were informed about the study and written information was provided. If the patient required time to consider participation, up to one hour was accepted. If the patient requested to discuss participation with a lay representative before a decision to participate was made, this was secured by a telephone call to the lay representative appointed by the patient if the representative was not present. When a written consent to participate was obtained the patient was included in the study.

### Method and data

We registered date and time of arrival and measurement, ear and rectal temperature in Celsius degrees, age, sex, use of hearing aid and if the patient had a medical or surgical complaint and any use of antipyretics and time of consumption. All information was obtained from the patients with no access to the patient file.

For the index test, we used the ear thermometer Braun Thermoscan Pro 4000®, released in 2006, where a heating element in the sensor warms the probe tip to just under normal body temperature to avoid cooling the ear canal, which has shown good correlation with the core temperature [3, 6]. For the reference rectal temperature an Omron Flex Temp Smart ®. Both devices were used routinely in the ED. To secure the quality of the instruments only one specific thermometer of each type was used and calibrated before and during the study. On each inclusion day, a double measurement of temperature in one patient was performed with both instruments to secure that the individual instrument measured the same temperature both times.

The research assistants, two medical students and a nurse, who was employed specifically for this purpose and had no relation to the ED staff, performed the ear temperature measurements and recorded the result without telling it to the patient. Since the measurements are not dependent on which side it is performed, the most convenient side was chosen [3, 8]. If the patient used hearing aid this was removed more than 5 min before the measurement. The assistant then performed the rectal measurement while the patient remained in the bed. The result of the rectal measurement was read by the research assistant and recorded accordingly. The research assistant was in this way not blinded for comparison of the results from the two measurements. At no point in the study the ED nurses were involved in the temperature measurements or had any influence on the registrations.

### Statistical analysis

Data were first collected in the Survey Xact ® electronic registration tool and transferred to STATA/IC 14.2 StataCorp, College Station Texas, USA for data purification and statistical analysis.

Since the definition of fever varies in literature from 37.6–38.3 °C [11] we used four different cut-points in our analysis: rectal temperature ≥ 37.5, 37.7, 38.0 and 38.3 °C with 38.0 °C as primary cut-point and calculated the sensitivity, specificity, predictive values, likelihood ratios and area under the curve (AUC) in a receiver-operator characteristics (ROC) diagram for the different cut-off points. The tympanic thermometers ability to predict the rectal temperature was analyzed with Bland-Altman analysis to calculate the prediction intervals. We performed subgroup analysis concerning age, time span from admission time and use of hearing aid.

### Ethics

As well the rectal measurement as the ear measurement of temperature were standard procedures in the ED and is well known to the public and used even at home by non-health professionals. The measurements might be slightly unpleasant, but represent no notable risk to the patient.

If the patients refused to participate, they received the usual examinations on arrival, including temperature measurements performed by the nurse assigned to the patient.

Approval from the Regional Committees on Health Research Ethics for Southern Denmark was waived after request (S-20160154). The study was registered with the Danish Data Protection Agency (16/39984).

## Results

### Baseline demographic and clinical characteristics of participants

**Fig. 1:**
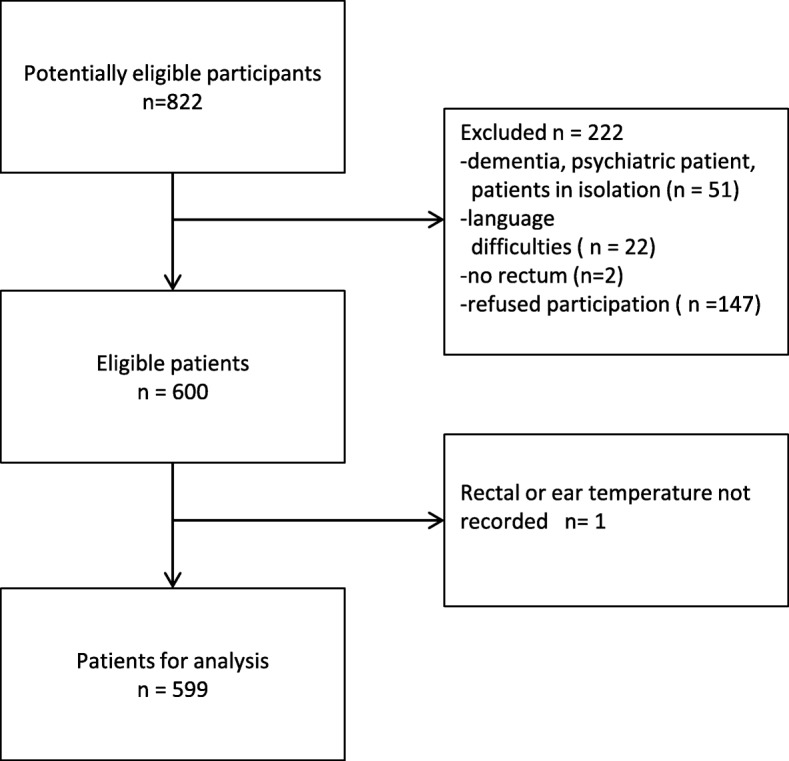
Recruitment and inclusion of patients

In a convenience sample of 40 randomly chosen days between December 2016–January 2017, we found 599 patients eligible for analysis (Fig. 1) with a mean age of 65.3 years, a median age of 69 years (IQR 54–77 years), 53% were women, 58% had medical reasons for admission and 42% were admitted due to surgical issues.

Among the patients, 33% had the study temperature measurement performed within the first hour after admission and 49% more than 12 h after the admission. The mean time interval between the TM temperature measurement and rectal temperature measurement was 0.7 min (95% CI: 0.6–0.8 min), 31% of the patients had taken an antipyretic medication on average 2.3 h (95% CI 2.1–2.5 h) before the temperature measurement, but none between the measurements; 8% used a hearing aid device.

#### Test results

**Fig. 2:**
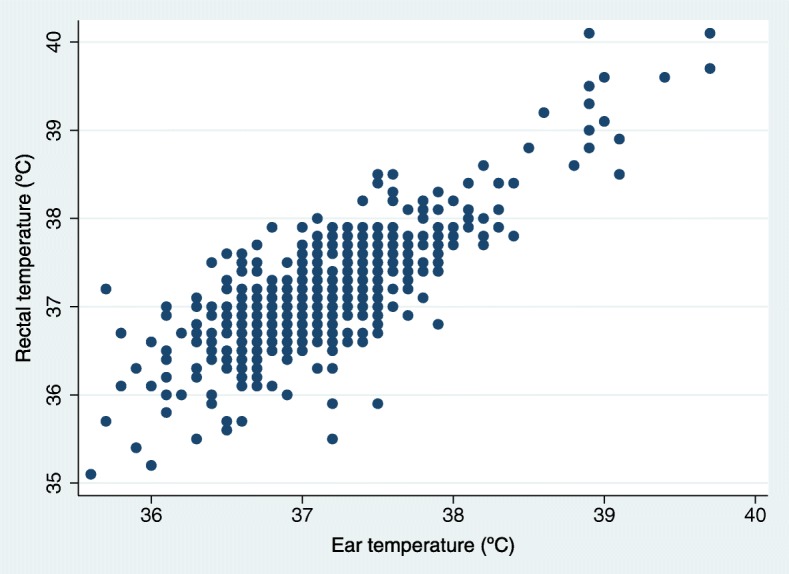
Scatter diagram of rectal and ear temperature measurements

The distribution of temperature measurements is visualized in the scatter diagram (Fig. 2). The mean ear temperature was 37.1 °C (SD 0.6 °C, range 35.6–39.7 °C) and the mean rectaltemperature 37.1 °C (SD 0.6 °C, range 35.1–40.1 °C).

Table 1 shows the TM thermometers ability to detect fever at the different cut- point for the rectal temperature. For all fever defining cut-points of rectal temperature, the sensitivity to detect a patient with fever using the ear thermometer varied between 68 and 70%, with negative predictive values (NPV) ranging from 90 to 99% and AUC from 0.88–0.97. Since the low sensitivity was a matter of concern, we further analyzed the TM thermometers ability to detect rectal temperatures of ≥38 °C. If the TM temperature was ≥37.5 °C, the sensitivity to detect patients with ≥38.0 °C rectally was 95% which raised to 100% for a rectal temperature of ≥38.3 °C, with corresponding NPV of 100%. The high sensitivity and NPV were at the expense of the specificity (82–83%) and positive predictive values (20–29%). A strategy of screening all patients with the TM thermometer and continue with rectal measurements for all patients having ear temperature ≥ 37,5 °C would result in 78% of all patients would not need rectal measurements, and 95% of all patients with temperature ≥ 38.0 °C would still be identified.

**Table 1:** Ear thermometers diagnostic performance at different cut-point for definition of fever

Temperature measurements							Screening test measures (%) (rectal measurement gold standard)								Test performance measures					
Rectal temperature			Ear temperature																	
fever definition	no. with fever	(%)	fever definition	no. with fever	(%)	TP	sensitivity	95% CI	specificity	95% CI	PPV	95% CI	NPV	95% CI	LHR(+)	95% CI	LHR(−)	95% CI	AUC	95% CI
≥ 37.5 °C	149	25	≥ 37.5 °C	133	22	101	68	60–75	93	90–95	76	68–83	90	87–92	9.53	6.71–13.6	0.35	0.27–.43	0.88	0.85–0.92
≥ 37.7 °C	98	16	≥ 37.7 °C	79	13	59	60	50–70	96	94–98	75	64–84	93	90–95	15	okt-24	0.41	0.33–0.53	0.92	0.89–0.95
≥ 38.0 °C	41	7	≥ 38.0 °C	39	7	26	63	47–78	98	96–99	67	50–81	97	96–99	27	15–49	0.38	0.25–0.56	0.96	0.93–0.98
≥ 38.3 °C	27	5	≥ 38.3 °C	22	4	19	70	50–86	99	99–100	86	65–97	99	97–99	134	42–426	0.30	0.17–0.53	0.97	0.95–0.99
≥ 38.0 °C	41	7	≥ 37.5 °C	133	22	39	95	84–99	83	80–86	29	22–38	99.6	98–100	5.65	4.64–6.88	0.11	0.04–0.28	nc	nc
≥ 38.3 °C	27	5	≥ 37.5 °C	133	22	27	100	87–100	82	78–85	20	14–28	100	99–100	5.31	4.48–6.3	0	nc	nc	nc

*nc* not calculated, *PPV* positive predictive value, *NPV* negative predictive value, *LHR(+)* likelihood ratio positive test, *LHR(−)* likelihood ratio negative test, *AUC* area under curve, *TP* True positive (True negative, false positive and false negative are all calculable by using TP, no. with rectal and ear fever information)

**Fig. 3:**
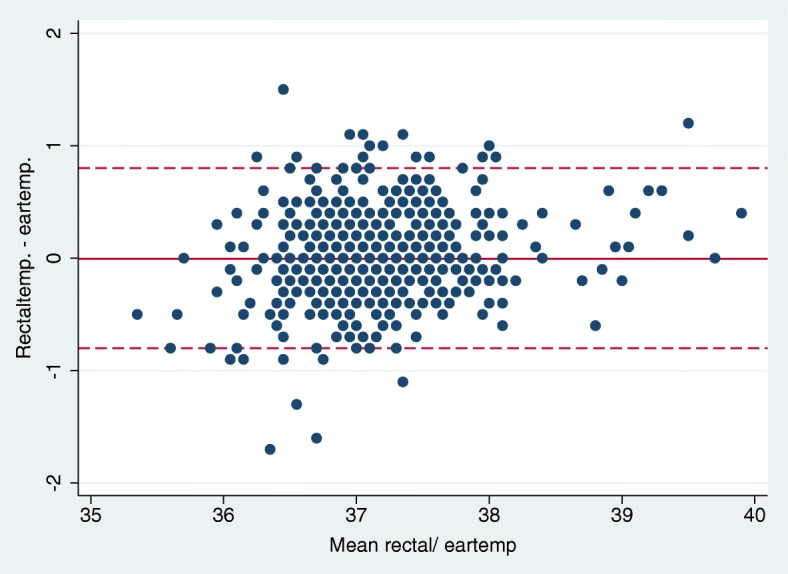
Bland Altman plot for TM and rectal temperatures

Table 2 reports the analysis of the ear thermometers ability to determine the exact temperature measured by rectal thermometers. The 95% limits of agreement were +/− 0.8 °C, (BA plot Fig. 3) and this did not change within the age groups, the duration in the hospital stay, hearing aids or if the analysis was restricted to ear temperatures between 36.0–37.9 °C.

**Table 2:** Bland- Altman analysis of temperature variation within various groups

					Bland-Altman comparison (°C)		
	no. of patients	method	mean temperature (°C)	95% CI (°C)	mean difference (°C)	95% CI	95% limits of agreement
total	599	ear	37.1	37.1–37.2	0.06	−0.03- 0.04	−0.8- 0.8
	599	rectal	37.1	37.1–37.2	reference		
Age groups							
18–39 years	54	ear	37.1	37.0–37.3	0.06	−0.2-0.04	− 0.8- 0.7
		rectal	37.1	36.9–37.2	reference		
40–59 years	134	ear	37.1	37.0–37.2	0.03	−0.07- 0.07	−0.8- 0.8
		rectal	37.1	37.0–37.2	reference	−0.04- 0.06	−0.8- 0.8
60–79 years	287	ear	37.1	37.0–37.2	0.01		
		rectal	37.1	37.1–37.2			
> 80 years	124	ear	37.1	37.0–37.1	0.02	−0.04- 0.08	−0.7- 0.7
		rectal	37.1	37.0–37.2	reference		
Hearing aid	48	ear	37.2	37.0–37.4	0.1	−0.2- 0.01	−0.8- 0.6
		rectal	37.1	36.9–37.3	reference		
Time after admission							
< 1 h	125	ear	37.2	37.1–37.4	0.1	0.01–0.2	−0.8- 1.0
		rectal	37.3	37.2–37.5	reference		
2–11 h	179	ear	37.1	37.1–37.2	0.07	−0.1- 0.02	−0.8- 0.7
		rectal	37.1	37.0–37.2	reference		
> 11 h	289	ear	37.0	37.0–37.1	0.01	−0.03- 0.06	−0.7- 0.8
		rectal	37.1	37.0–37.1	reference		
Ear temperature 36.0–37.9 (°C)	550	ear	37.0	37.0–37.1	0.004	−0.03- 0.04	−0.8- 0.8
		rectal	37.0	37.0–37.1	reference

No adverse events were observed with the use of ear thermometers.

## Discussion

Our study showed that the examined device for TM temperature measurement was useful to exclude fever, defined as ≥38.0 °C in an ED population having a 7% prevalence of fever if the cut-point for the TM thermometer was set to 37.5 °C. A strategy of using the TM thermometer as a screening tool and continue with rectal measurements for all patient with ear temperature of ≥37.5 °C would identify 95% of all patients with true rectal fever of ≥38.0 °C, and reduce the number of patients who needed a rectal measurement to 22%.

To estimate the exact rectal temperature using the TM thermometer would result in a wide 95% limitation interval of +/− 0.8 °C independent of age, time after arrival, use of hearing aid or even if restricted to normal ear temperatures.

For many years clinicians have sought for faster, more convenient alternatives to a rectal measurement of the temperature in hospitalized adult patients. The studies of tympanic membrane temperature devices have so far shown disappointing results or generally been weakened by simple statistical analysis, special patient groups, like children or intensive care patients or use of old technologies.

Our results, based on 599 adult patients in an ED with use of a new generation technology, do not support the use of tympanic devices for exact measurement of temperature since the inaccuracy of around +/− 0.8 °C exceeds a clinically acceptable variation. However, for screening purpose our results showed that the used TM device was able to detect fever of ≥38.0 °C with a sensitivity of 95%, when using a TM cut-point of 37.5 °C.

Two other studies have examined the adult ED population using one of the new generation of TM thermometers. A study from Bijur et al., US (2016) [7], including 987 patients with a prevalence of fever (> 38.0 °C) of 29%, using the same device as we did, showed that a TM cut-point of 37.5 °C resulted in a 91% sensitivity and 90% specificity, but a − 1.2-06 °C limits of agreement, far beyond a clinically acceptable variation. The researchers concluded that lowering the TM cut-point to 37.5 °C was a viable option for the use of TM measurement to screen for fever. Our results are in alignment with this study.

The other ED study, from Barnett et al., US (2011) [8] of 455 adult ED patients with a prevalence of fever of 19% (> 38.0 °C), used another TM device (First Temp Genius II, model 3000 A ®). They found a – 0.9-1.2 °C (converted from Fahrenheit) limits of agreement, a 74% sensitivity and 86% specificity to detect fever, using a cut-point of 38.0 °C on the TM. This study sought for an optimal cut-point yielding the highest sensitivity and specificity. With a cut-point of 37.9 °C an 80% sensitivity and 80% specificity was obtained. The researchers suggested that rectal thermometry should still be measured.

Our results concerning the limits of agreement corroborate both studies: the TM thermometers are still not accurate enough to measure the exact temperature within a clinically acceptable agreement with the rectal temperature.

Concerning the use of the TM as a screening for fever, our results is in agreement with the study from Bijur et al., and we recommend the TM to be used as a screening tool to detect fever using a TM cut-point of 37.5 °C. The study from Barnett et al. tried to find an optimal cut-point by maximizing the combined sensitivity and specificity. In the ED we find it more important to identify the true febrile patients, i.e. to maximize the sensitivity to more than 90%, at the expense of the specificity [7]. Since the study of Barnett actually increased the sensitivity of 74 to 80% by just lowering the TM cut-point by 0.1 °C, it is likely that their results are not far from the Bijur et al. study [7] or from our results concerning a TM cut-point of 37.5 °C .

The use of the current TM technology was not influenced by age, hearing devices or time since arrival. This finding has not been investigated in other studies using the new generation TM technologies but encourages us to believe are robust and the technology is useful in a range of different situations and in other in-hospital departments. Since the prevalence of fever in the Bijur study [7] was 29% and in ours only 7% we believe that the use of TM as a screening tool for fever is useful within a wide range of patient groups.

The clinical implication of our findings is that the new generations of TM devices, studied by our and other research groups [7, 8] can be used as screening tools to detect fever (defined as ≥38.0 °C rectally). If the TM thermometer measures temperature ≥ 37.5 °C, the health staff should proceed with a rectal measurement to obtain the exact temperature and not rely on the TM measurement. In our study, this approach would mean that 78% of the patients would not be in need of a rectal measurement, and 5% of the patients with a fever ≥38.0 °C would not be identified, but all with a temperature ≥ 38.3 °C would be. The results also imply that proper training of the staff and maintenance of the equipment is required.

The strengths of our study are first the size, being the second largest study in its field and the only study analyzing subgroups of patients. Secondly we used recommended statistical methodology. There are some limitations to the study as well. First, the choice of rectal temperature as a reference temperature, which was chosen as the most accepted surrogate measure of the core temperature and in accordance with other published studies, is debatable [12]. Secondly, we primarily chose 38.0 °C as the definition of fever, acknowledging that the temperature varies with by the time of day and gender. Thirdly we used three research assistants who were trained, employed and continuously supervised for this assessment only and who controlled the instruments regularly. While we consider it a strength to use independent research assistants concerning the internal quality of the study, we acknowledge that implementing the use of TM technology in a busy ED in hands of several staff members might reduce the quality of the measurements obtained. Since the TM devices are simple to use and already widespread in many EDs we do not believe this to be a major problem. Fourthly we did not reach the estimated sample size in two of the subgroups (hearing devices and age 18–39 years). Finally, a single center ED might not represent all other patient categories or EDs and we did not include patients less than 18 years. However we have performed a similar study in the pediatric department of our hospital including 995 children which resulted in the same conclusions as the present study [13]. We find our results similar to other ED studies with different prevalence of fever and our subanalysis of age, admission time and use of hearing aid did not influence the temperature measurements.

## Conclusion

The examined type of the new generation of TM thermometers is able to detect fever, defined as ≥38 °C rectally in an adult ED population by using a TM cut-point of 37.5 °C, but not to measure the exact temperature. Used in this way around a fifth of the patients will still be in need of a rectal temperature measurement, but less than 5% with fever ≥38.0 °C and will remain undetected and none with fever ≥38.3 °C. Age, admission time and use of hearing aid did not influence the temperature measurements. Development of devices for exact measurement of temperature is still warranted and future clinical studies are needed to test the accuracy of every new generation of thermometers.

## Abbreviations

AUC: Area under the curve
ED: Emergency department
NPV: Negative predictive value
ROC: Receiver-operator characteristics
TM: Tympatic membranes
